# Social Networking Site Usage and Its’ Impact on Depressive Symptoms among Older Men and Women in South Korea

**DOI:** 10.3390/ijerph17082670

**Published:** 2020-04-13

**Authors:** Gyeong-Suk Jeon, Kyung-Won Choi, Kwang-Sim Jang

**Affiliations:** 1 Department of Nursing, Division of Natural Science, Mokpo National University, Muan-gun 58554, Korea; sookie@mokpo.ac.kr; 2 Department of Nursing, Korea National University of Transportation, Chungbuk 27909, Korea; 3 Department of Nursing, Sehan University, Youngam 58447, Korea; ksjang@sehan.ac.kr

**Keywords:** social networking sites, social media, depressive symptoms, older adults, gender

## Abstract

*Background*: We examined the rate of social networking site (SNS) usage and the relationship between SNS usage and depressive symptoms among older men and women in South Korea. *Methods*: The data were obtained from the Living Profiles of Older People Survey (LPOPS), which comprises a nationally representative sample of non-institutionalized Korean older adults living in the community. A total of 10,073 older persons (4286 men and 5787 women) were included in the analysis. *Results*: A total of 26.0% of the total study population reported to use social network sites. Of the men and women, 32.6 and 21.1%, respectively, used SNS. A higher educational level and higher equivalent household income, lower number of chronic illnesses, no limitation in daily activities, living with others, and having good relationships with friends and neighbors were associated with higher SNS usage among older men and women. SNS usage was significantly associated with reduced depression scores in older men, but not in women. *Conclusions*: The difference in SNS usage between older men and women might be partially explained by differential exposure to digital technologies and disadvantages in education and economic opportunities. Education programs for digitalization and opportunities to engage with technology need to be provided.

## 1. Introduction

With the global elderly population rapidly increasing and the expected average life span continuing to rise, the topic of healthy and active aging is gaining attention. Previous studies have shown that the physical and psychological health of older adults depends on the maintenance of social networks and relationships [1,2,3,4]. Emotional support from social networks can help keep older adults functionally capable [1], while the absence of social participation and social connections increases the sense of loneliness, along with the risk of depression and functional decline in the older people [2,3,4]. Mobility limitations, geographical distance to family or friends, and other obligations, such as caregiving, can prevent older adults from engaging in these social contacts. Evidence suggests that the use of the Internet and social networking sites (SNSs, also known as “social media”) among older adults increases the frequency of social contact, enhances social interaction, and helps cope with stress and depression [5,6]. Social networking sites (SNSs) are the latest web-based communication tool allowing for individuals to create a public or semi-public profile, interact with other users within the system, and view the list of connections that are shared with others [7]. Online channels typically enable users to connect with others, regardless of geographical location and time, create opportunities to share topics of interest, provide emotional support to those in need, as well as keep in touch with friends and family. Consequently, SNSs can play an important role in connection and communication, which suggests that SNS use might to help maintain preexisting social networks, facilitate social interaction, and reduce the risk of depression in older people.

Some studies investigated SNS use and the impact on the mental health of older adults [8,9,10,11,12,13]. The results from these studies indicate that SNS usage in older population is associated with higher levels of social role satisfaction [8] and maintenance of social connectedness [9,10], and they have a positive impact on their psychological health by connecting them to family, friends, and relevant information, and reducing loneliness [8,11,12]. Although the results are inconsistent, a few studies suggested that there was significant gender difference in the influence of SNS use on health outcome [9,12]. Older men showed higher levels of life satisfaction with SNS usage than older women [12]. Meanwhile, a longitudinal study showed that male users reported more loneliness than female users [9].

Patterns of social networks and social support have been shown to vary according to gender [14], and these patterns appear to be fairly consistent across an adult’s life span [15,16]. Gender theory holds that women differ from men in terms of their engagement in communication, their enjoyment of communication, and the extent to which they can be influenced by others. In addition, access, use, and ownership of digital tools are not gender-neutral, and the benefits of the digital revolution have not been equally shared between genders [17]. Several explanations for this gender-based digital exclusion have been suggested, including barriers to access, education, lack of technological skills, and socio-cultural norms [18]. Given these trends, men and women should be separately assessed when examining the use and impact of internet-based communication tools, such as SNSs, for social support. However, there is a scarcity of research focusing specifically on gender difference in the relationship with mental health, as well as SNS usage, independently, for men and women.

In South Korea, a country that is known for its advanced Information Technology, 90.3% of the population of 2017 use the Internet, highlighting its central role in everyday life for most Koreans [19]. Among internet users in South Korea, 68.2% are engaged with SNSs; Although uptake in adults aged 60 years and older is relatively low (37.5%), when compared to SNSs use of older adults in the past (11.1% in 2013) [20], SNSs have become increasingly popular among older age groups in South Korea, with the adoption rates nearly triple among adults aged 60 and older. With increasing use of SNSs among older adults in South Korea, little is known regarding the characteristics of older adult users and its impact on their mental health.

This study aimed to investigate the characteristics of SNS users and the effect of SNS usage on depressive symptoms among older men and women to address these research gaps, using a nationally representative sample of older adults in South Korea.

## 2. Materials and Methods

### 2.1. Design and Study Population

This study used cross-sectional data from the 2017 Living Profiles of Older People Survey (LPOPS), which is conducted every three years by the Korean Ministry of Health and Welfare. LPOPS examined a nationally representative sample of community dwelling older adults (aged 65 years and older) who lived within 16 regions (seven metropolitan and nine provincial regions) in both urban and rural areas of South Korea. A nationwide probability sample of non-institutionalized older adults was selected while using a stratified two-stage cluster sample design. All of the participants provided written informed consent. A total of 10,073 older adults completed interviews that were conducted by trained interviewers. The participants who responded by proxy and those with missing data relevant to this analysis (i.e., depressive symptoms and income) were excluded. Following exclusions, 4286 men and 5787 women aged 65 years or older were included and analyzed.

### 2.2. Assessment and Measurements

SNS usage. To determine SNS usage, the participants were asked, “Do you use social networking sites such as Band, Kakao Talk, Twitter, Facebook, Instagram, or Telegram Talk?” Those who responded “yes” were considered to be SNS users.

Depressive symptoms. Depressive symptoms were evaluated using the South Korean Geriatric Depression Scale-Short Form (SGDS-K). The Geriatric Depression Scale (GDS) was developed by Yesavage and Sheik [21] and translated into South Korean by Bae and Cho [22]. The 15-item SGDS-K has been shown to have equal validity and it is less affected by education, current employment, living accommodation, and chronic diseases when compared to the 30-item K-GDS [23]. The 15 items included five positive and 10 negative feelings for the previous week, and each coded as negative (1) or positive (0), with total scores ranging from 0 to 15. The five items with positive feelings were inverted, and higher summed scores indicate greater depression severity. A SGDS-K score of 8 or higher was used as the threshold for major depressive disorder. The SGDS-K has demonstrated good reliability (Cronbach’s alpha of 0.90) and validity [22].

Health variables Numbers of chronic illnesses were assessed while using participants’ self-reported total number of physician-diagnosed conditions, such as hypertension, diabetes, etc. The participants were categorized in three groups, as having none, one, or two or more chronic illnesses. Limitations in Activities of Daily Living (ADL) were assessed using the South Korean versions of the Activities of Daily Living (K-ADL) and the Instrumental Activities of Daily Living scale (K-IADL). The respondents were asked whether they required assistance while performing seven daily activities (i.e., dressing, washing their face/shampooing/teeth-brushing, bathing, eating meals, getting up and moving out of their room, using the toilet, and controlling urination and bowl movements), and ten different instrumental activities (i.e., personal hygiene and grooming, housekeeping, preparing meals, making and receiving phone calls, managing money, taking medications as prescribed, using transportation, shopping, and doing laundry). The K-ADL/IADL scores were categorized as having no limitations in daily activities (K-ADL/IADL = 0) or having limitations in daily activities (K-ADL/IADL ≥ 1).

Other covariates Age (65–74, 75–84, 85, or over), area of residence (urban or rural), living arrangement, education, economic activity, annual household income, and relationship satisfaction with friends and neighbors were included as covariates. The living arrangements were grouped into four categories: living alone, living with spouse only, living with children, or living with others (i.e., grandchildren). Education levels were classified as college or higher education, high school, middle school, and elementary school or uneducated. Economic activity was classified as “yes” or “no”. The total annual household income was divided by the square root of the number of household members and equivalent household income was categorized into quartiles (1st 25%; 2nd 25%; 3rd, 25%; 4th 25%). Relationship satisfaction with friends and neighbors was rated on a scale from 1 (very good) to 5 (very bad) in response to the question, “How would you rate your relationship with your friends and neighbors?” Based on their responses, relationship satisfaction was categorized as “good” (score of 1–2), “fair” (score of 3), and “poor” (score of 4–5).

### 2.3. Statistical Analysis

The data were expressed as the frequencies, weighted proportions, and means ± standard deviation (SD) of the baseline characteristics by gender. Chi-square tests were used to compare the distribution of these frequencies between older men and women (Table 1). Logistic regression analysis was conducted to assess the impact of SNS usage on depressive symptoms (Table 2). Wald chi-squared tests were used to compare the regression coefficients of gender-specific models to assess the differences between gender groups [24]. We evaluated the possible multicollinearity between covariates, such as SNS usage and the relationship satisfaction with friends and neighbors by correlation analysis and collinearity statistics tests (tolerance and variance inflation factor tests), as suggested for logistic regression [25]. No significant collinearity was detected between any of the covariates. All of the statistical tests were conducted using IBM SPSS software v.23.0 for Windows (IBM Corp., Armonk, NY, USA). The Ethics Review Board of the South Korea National University of Transportation approved this study, with which the researchers were affiliated (KNUT IRB 2020–2).

## 3. Results

Table 1 summarizes participants’ characteristics and SNS usage by gender. Among study participants, the mean age was similar for women (73.61 years) and men (74.07 years). More older women (33.6%) than older men (10.9%) lived alone, and the percentage of elementary school or uneducated was higher among older women (33.8%) than older men (10.2%). More older men (38.3%) participated in economic activity than older women (25.5%). The majority of older men and women had two or more chronic illnesses (64.1% and 79.6%, respectively), and had no limitations in daily activities (88.4% and 73.0%, respectively). Over half of older men and women had good relationships with friends and neighbors (60.8% and 59.8%, respectively).

Among older adults, 32.6% of men and 21.1% of women reported using SNSs. The percentage of SNS use was higher among men living in urban areas. Older men who lived with someone were more likely to use SNSs than those who lived alone. A higher educational level, higher equivalent household income and participation in economic activity were all associated with SNS usage. Older men who had no limitations in daily activities, a lower number of chronic illnesses, and good relationships with friends and neighbors showed a higher frequency of SNS use than their counterparts. Among older women, the percentage of SNS usage was also higher among those that were living in urban areas and those living with someone. A higher educational level and higher equivalent household income, lower number of chronic illnesses, no limitation in daily activities, and having good relationships with friends and neighbors were associated with higher SNS usage among older women. However, unlike older men, older women who had no economic activity showed higher SNS usage than those who undertake have economic activity.

The left-hand panel of Table 2 reports the prevalence of depressive symptoms according to each interest variables by gender. Depressive symptoms were higher in older women (15.6%) than older men (10.4%) (*p* < 0.01). Older men (3.9%) and women (6.5%) who used SNSs both reported lower depressive symptoms than their counterparts (men 13.6%, women 18.0%) (*p* < 0.01). As expected, being older, living alone, having low education and low equivalized household income, having no economical activity, having chronic illness and daily activity limitation, and having poor relationship satisfaction with friends and neighbors were significantly correlated with elevated risk of depressive symptoms among South Korean older men and women (*p* < 0.01). In addition, significant differences were found between men and women in the descriptively examined depressive symptoms with the same levels of living arrangement, education attainment, chronic illness, daily activities limitation, and relationship with friends and neighbors.

The right-hand panel of Table 2 presents the results of the multivariate logistic regression analysis, as odds ratios (ORS) and 95% confidence intervals (CIs), assessing the relationships between SNS usage and depressive symptoms after controlling for covariates. The absence of SNS use was associated with a higher the risk of depressive symptoms among older men (OR = 1.59, 95% CI = 1.13–2.23), but not older women (OR = 1.28, 95% CI = 0.96–1.69). However, gender difference was not significant in effect of SNS usage on depressive symptoms by Wald chi-square tests. Living alone was associated with depressive symptoms in men (OR = 1.61, 95% CI = 1.18–2.18) and women (OR = 1.48, 95% CI = 1.21–1.81). Having elementary education attainment or being uneducated were significantly associated with depressive symptoms in both men (OR = 1.67, 95% CI = 1.16–2.40) and women (OR = 1.52, 95% CI = 1.11–2.07). In addition, older men and women being in 4th 25% of equivalent household income were twice as likely to report depressive symptoms. Being separated from the labor market was significantly associated with depressive symptoms in both genders. Older men and women having two or more chronic illness and daily activities limitation were at much higher risk of depressive symptoms than were each reference groups. Having poor relationship satisfaction with friends and neighbors was strongly associated with depressive symptoms in older men (OR = 4.76, 95% CI = 3.60–6.31) and women (OR = 4.62, 95% CI = 3.74–5.72).

## 4. Discussion

The objective of this study was to examine the characteristics of SNS users and the relationship between SNS usage and depressive symptoms among older men and women in South Korea. The results showed that there are gender differences in SNS usage and that socioeconomic factors were associated with SNS use in both older men and women. In addition, this study showed that SNS usage was associated with reduced depression scores among older men but not women.

The observed gender difference in SNS usage between men (32.6%) and women (21.1%) might be explained by differences in their socioeconomic conditions. In this study, older women showed higher rates of living alone, elementary or uneducated, lowest household income, no economic activity, and more chronic illnesses when compared to older men. The digital gender divide, which refers to gender differences in resources and capabilities to access and effectively use information and communication technology (ICT), might have contributed to these findings [26]. Despite these findings, the SNS usage gap between older men and women in South Korea might be gradually closing. This study showed that the difference in percentage of SNS usage between older men and women is decreasing according to increasing age (presented in Table 1). Korea national statistics with representative data also show that gender gaps in SNS use in overall population have diminished gradually (3.8% in 2016, 2.5% in 2017, and 1.8% in 2018) [27]. This expectation that there is a reduction of gender gap in usage of SNS among older Koreans could be explained the following reasons. The digital environment of Korea, which has few barriers to accessing the Internet thus most individuals could connect digital devices, could also offer opportunities for increased digital device use among older women in South Korea. With internet connection being available across the country, the rate of smartphone users (i.e., individuals who have used wireless internet via a smartphone) in people aged 50 and less years is over the 97% [19]. This means that, unlike older adults that lack material resources and digital skills, access to internet and digital skills have reached near gender parity at least in younger generations, with women accounting for an increasing percentage of internet users, and no gender difference in SNS usage (68.0% of men and 68.4% of women) [19]. Second, the reduction of gender inequality of South Korea for recent decades also increase opportunities for women in the design, development, production, and use of digital technologies, which might continue to narrow the digital gender gap among future Korean older adults. Actually, South Korea maintained cultural characteristics with traditional norms governing the household division of labor by patriarchy and Confucianism, and it has consistently ranked among the highest gender inequality the world [28]. By Confucian philosophy, Korean men and women were required to strictly follow the guiding principle of gender relations, *Namjon yobi*, which means, ‘Men should be respected and women should be lowered’. Not only subordinate status in family and society, but rigorous standards of feminine modesty and chastity had an overwhelmingly negative impact on the lives of Korean women. As a result, Confucianism has been blamed for the strong discrimination against women in Korean society [29]. Although Confucian ethics has pervaded everyday Korean life, and has been reflected in many practices, it has been weakened gradually with industrialization and modernization. According to the 2007 Global Gender Gap Report [24], South Korea ranked 97th out of 128 countries for gender equality. However, in 2019, South Korea has made some progress in promoting gender equality, achieving near-parity in health and survival, and increasing their rank to 108th globally out of 153 countries [30]. While gender inequality remains prevalent, there have been improvements in several dimensions, including political empowerment (ranked 79th), economic participation (127st), and education attainment (101th).

The results also showed that individuals who were older, had a lower educational level and household income, and lived in rural areas had lower rates of SNS use than their counterparts, among both older men and women. These findings are supported in the literature on internet usage, with studies from several countries identifying a similar phenomenon and concluding that offline structural inequalities, such as those that are related to income and education, are reflected in the ways people engage with digital technologies [31,32]. These structural inequalities can result in having less access to digital technologies, making less use of them, and/or gaining less benefit from them [17].

Although gender differences were not statistically significant in the effect of SNS use on depressive symptoms after control for covariates, SNS usage was associated with a 60% decrease in the risks of depressive symptoms among older men. While some researchers have suggested that the effects of SNS usage on mental health in older adults are inconclusive [33,34], a recent Chinese study revealed that older men exhibit higher life satisfaction when compared to women when they use SNSs [12]. The positive impact of SNS usage on depressive symptoms among older men might be explained by the opportunity that SNS usage provides for social connectivity. Since men typically experience a dramatic reduction in the size of their social network and often have difficulty making new social relationships after their retirement, a greater number of older men report moderate to high levels of social isolation [35]. Moreover, older men in patriarchal societies, such as South Korea and China, are more likely to have partner-centered networks and lack other social contacts, including close friends [36,37]. The participation rates in community senior centers and welfare centers were higher in older women (26.4% and 9.8%, respectively) than older men (18.4% and 8.5%, respectively) [38]. Thus, SNS use might mitigate for lack of social contacts among older men, resulting in a positive effect on depressive symptoms. Unlike men, women tend to have larger, denser and more diverse social networks [39], and relationships with their friends are typically oriented to affection, supportiveness, and empathic understanding [40,41]. Through these social networks, women experience improved social contact and emotional support [42], and maintain more friendships as they get older [43]. Older women who maintain their social network using traditional forms of communication, such as face-to-face conversations and telephone calls, might be less inclined to use SNSs.

There were several limitations to this study. First, the analysis relied on cross-sectional data, which precluded the ability to assess causal relationships. Further analyses of longitudinal data are necessary to clarify the relationships between SNS usage and depressive symptoms among older adults. Second, the duration and frequency of SNS use were not considered in this study. Finally, the possibility that the effect of SNSs usage on depressive symptoms is underestimated cannot be ruled out, because the relationship satisfaction with friends and neighbors that could be an endogenous variable was included in multiple logistic regression of this study as a covariate. Despite these limitations, this study addressed several gaps in the literature. First, this study used a nationally representative sample that was weighted by census estimates, increasing the generalizability of these findings. Second, the results show that there are significant differences in SNS use according to socioeconomic status and gender, and that the effect of SNSs on depressive symptoms also varied by gender. These findings suggest that further attention is needed in order to address the digital gender gap, and that interventions using SNSs might help to prevent depressive symptoms in older adults.

## 5. Conclusions

This study examined the characteristics of SNS users and the impact of SNS use on depressive symptoms among older men and women in South Korea. In this nationally representative sample, structural inequalities were observed in SNS usage rates in both older men and women, and SNS usage was significantly associated with reduced depression scores in older men. The observed gender difference might be partially explained by differential exposure to digital technologies and persisting disadvantages in education and economic opportunities. Thus, diverse education programs for digitalization and opportunities to engage with technology should be provided. The accessibility of the Internet might play an increasing role in SNS usage and its impact on depressive symptoms in older adults. Further longitudinal research is necessary in order to evaluate the relationship between SNS usage and depressive symptoms among older men and women.

## Figures and Tables

**Table 1:** Distribution of Socioeconomic and Health Characteristics, and Prevalence of Social Network Site Usage among Men (*n* = 4286) and Women (*n* = 5787) Older Adults aged 65 years or over in the 2017 South Korean Older People Living Conditions Profile Survey.

	Men *n* (%) or Mean ± SD		Women *n* (%) or Mean ± SD			All *n* (%) or Mean ± SD		*Social Network Site Usage*			
								Men	Women		All
Prevalence of social network site usage								32.6	21.1	^‡^	26.0
Age	73.61	±6.34	74.07	±6.67		73.87	±6.54	**	**		**
65–74	2536	59.2	3313	57.2	^‡^	5849	58.1	43.2	32.7	^‡^	37.2
75–84	1468	34.3	1982	34.2		3450	34.2	18.6	6.9		11.9
85+	282	6.6	492	8.5		774	7.7	9.9	1.2		4.4
Residency area								**	**		**
Urban	2930	68.4	3993	69.0		6923	68.7	36.1	24.5	^‡^	29.4
Rural	1356	31.6	1793	31.0		3149	31.3	25.1	13.6		18.5
Living arrangement								**	**		**
Couple only	2778	64.8	2130	36.8	^‡^	4908	48.7	31.9	26.9	^‡^	29.7
With children	870	20.3	1501	25.9		2371	23.5	36.1	19.7		25.7
With others	170	4.0	209	3.6		379	3.8	44.7	24.9		33.8
Alone	468	10.9	1946	33.6		2414	24.0	26.3	15.6		17.6
Education								**	**		**
College or more	1620	37.8	897	15.5	^‡^	2517	25.0	53.5	59.0	^‡^	55.5
High school	919	21.4	792	13.7		1711	17.0	30.6	39.1		34.5
Middle school	1310	30.6	2141	37.0		3451	34.3	17.3	16.3		16.6
Elementary or uneducated	436	10.2	1956	33.8		2392	23.8	5	1.8		2.4
Equivalent household income ^a^								**	**		**
1st 25%	1216	28.4	1303	22.5	^‡^	2519	25.0	52.5	38.8	^‡^	45.5
2nd 25%	1121	26.2	1405	24.3		2526	25.1	34.3	25.9		29.7
3rd 25%	1034	24.1	1465	25.3		2499	24.8	20.4	14.2		16.8
4th 25%	915	21.3	1613	27.9		2528	25.1	17.8	9.0		12.2
Economic activity								**	*		**
Yes	1642	38.3	1475	25.5	^‡^	3117	30.9	36.3	19.2	^‡^	28.2
No	2644	61.7	4311	74.5		6955	69.1	30.3	21.8		25
Chronic illness								**	**		**
None	628	14.6	428	7.4	^‡^	1056	10.5	42.4	38.9	^‡^	41
One	909	21.2	755	13.0		1664	16.5	36.9	29.4		33.5
Two or more	2750	64.1	4604	79.6		7354	73.0	29	18.1		22.2
Limitation in daily activities								**	**		**
No	3789	88.4	4226	73.0	^‡^	8015	79.6	36.0	27.5	^‡^	31.5
Yes	496	11.6	1560	27.0		2056	20.4	6.7	4.0		4.6
Relationship satisfaction with friends/neighbors								**	**		**
Good	2608	60.8	3461	59.8		6069	60.3	41.3	27.7	^†^	33.6
Fair	1158	27.0	1668	28.8		2826	28.1	20.8	13.2		16.3
Poor	520	12.1	657	11.4		1177	11.7	15.2	6.5		10.4

* *p* < 0.05, ** *p* < 0.01, for difference among different levels of each variable; ^†^ *p* < 0.05, ^‡^ *p* < 0.01 for difference between men and women. ^a^ Monthly household income was divided by the square root of the number of household members and equivalized household income was calculated into quartile.

**Table 2:** Prevalence and Odds Ratio (95% Confidence Interval) ^b^ for Depressive Symptoms among Men (*n* = 4286) and Women (*n* = 5787) Older Adults Aged 65 Years or Over in the 2017 South Korean Older People Living Conditions Profile Survey.

	Depressive Symptoms				Men			Women		
	Men	Women		Total	OR	95% CI		OR	95% CI	
*N* =	4286	5787		10,073						
Prevalence of depressive symptoms	10.4	15.6	^‡^	13.4						
Social network site usage	**	**		**						
Yes	3.9	6.5		5.1	1			1		
No	13.6	18.0		16.3	1.59	1.13	2.23	1.28	0.96	1.69
Age	**	**		**						
65–74	7.7	11.8		10.0	1			1		
75–84	13.5	19.6		17.0	1.07	0.84	1.36	0.95	0.80	1.14
85+	18.8	25.2		22.9	0.87	0.59	1.30	0.80	0.61	1.06
Residency area		*		**						
Urban	11.0	16.3		14.1	1			1		
Rural	9.3	13.9		11.9	0.96	0.75	1.24	0.84	0.70	1.00
Living arrangement	**	**	^†^	**						
Couple only	9.2	10.4		9.7	1			1		
With children	10.1	16.7		14.3	1.55	1.12	2.13	1.56	1.23	1.96
With others	10.0	14.4		12.4	1.69	0.94	3.03	1.20	0.77	1.87
Alone	18.4	20.4		20.0	1.61	1.18	2.18	1.48	1.21	1.81
Education	**	**	^†^	**						
College or more	6.4	8.0		7.0	1			1		
High school	10.7	11.2		10.9	1.36	0.98	1.88	1.30	0.92	1.85
Middle school	11.9	13.5		12.9	1.40	1.04	1.88	1.25	0.92	1.69
Elementary or uneducated	20.4	23.0		22.6	1.67	1.16	2.40	1.52	1.11	2.07
Equivalent household income ^a^	**	**		**						
1st 25%	4.6	9.2		7.0	1			1		
2nd 25%	7.0	12.0		9.8	1.32	0.89	1.95	1.24	0.95	1.62
3rd 25%	13.2	15.8		14.7	1.95	1.32	2.86	1.69	1.29	2.23
4th 25%	19.1	23.7		22.0	2.62	1.77	3.87	2.20	1.67	2.90
Economic activity	**	**		**						
Yes	3.9	8.3		6.0	1			1		
No	14.5	18.1		16.7	2.32	1.71	3.13	1.87	1.50	2.32
Chronic illness	**	**	^†^	**						
None	3.5	2.6		3.1	1			1		
One	6.4	5.0		5.8	1.37	0.80	2.34	1.95	0.97	3.91
Two or more	13.3	18.5		16.6	2.41	1.51	3.84	5.96	3.21	11.06
Limitation in daily activities	**	**	^†^	**						
No	7.2	10.6		9.0	1			1		
Yes	34.9	29.2		30.5	3.30	2.55	4.27	2.02	1.70	2.41
Relationship satisfaction with friends/neighbors	**	**	^†^	**						
Good	5.0	8.8		7.1	1			1		
Fair	12.4	20.6		17.3	1.73	1.33	2.25	2.15	1.80	2.56
Poor	33.3	38.8		36.4	4.76	3.60	6.31	4.62	3.74	5.72

* *p* < 0.05, ** *p* < 0.01, for difference among different levels of each variable; ^†^ *p* <0.05, ^‡^ *p* < 0.01 for difference between men and women. ^a^ Monthly household income was divided by the square root of the number of household members and equivalized household income was calculated into quartile. ^b^ 95% confidence interval means that if we were to take 100 different samples and compute a 95% confidence interval for each sample, approximately 95 of the 100 confidence intervals will contain the true mean value.
